# Targeting Protein Kinases in Blood Cancer: Focusing on CK1α and CK2

**DOI:** 10.3390/ijms22073716

**Published:** 2021-04-02

**Authors:** Zaira Spinello, Anna Fregnani, Laura Quotti Tubi, Livio Trentin, Francesco Piazza, Sabrina Manni

**Affiliations:** 1 Department of Medicine, Hematology Section, University of Padova, Via N. Giustiniani 2, 35128 Padova, Italy; zaira.spinello@unipd.it (Z.S.); anna.fregnani@studenti.unipd.it (A.F.); laura.quottitubi@gmail.com (L.Q.T.); livio.trentin@unipd.it (L.T.); 2 Veneto Institute of Molecular Medicine, Via G. Orus 2, 35129 Padova, Italy

**Keywords:** protein kinase CK1, protein kinase CK2, blood tumors, survival/stress signaling, non-oncogene addiction

## Abstract

Disturbance of protein kinase activity may result in dramatic consequences that often lead to cancer development and progression. In tumors of blood origin, both tyrosine kinases and serine/threonine kinases are altered by different types of mutations, critically regulating cancer hallmarks. CK1α and CK2 are highly conserved, ubiquitously expressed and constitutively active pleiotropic kinases, which participate in multiple biological processes. The involvement of these kinases in solid and blood cancers is well documented. CK1α and CK2 are overactive in multiple myeloma, leukemias and lymphomas. Intriguingly, they are not required to the same degree for the viability of normal cells, corroborating the idea of “druggable” kinases. Different to other kinases, mutations on the gene encoding CK1α and CK2 are rare or not reported. Actually, these two kinases are outside the paradigm of oncogene addiction, since cancer cells’ dependency on these proteins resembles the phenomenon of “non-oncogene” addiction. In this review, we will summarize the general features of CK1α and CK2 and the most relevant oncogenic and stress-related signaling nodes, regulated by kinase phosphorylation, that may lead to tumor progression. Finally, we will report the current data, which support the positioning of these two kinases in the therapeutic scene of hematological cancers.

## 1. Introduction

Protein kinases cover a crucial role in cell biology by catalyzing protein phosphorylation. Phosphorylation is a post-translational modification (PTM) critical for the regulation of different cellular functions, such as proliferation, cell cycle, apoptosis, motility, growth, and differentiation. Perturbations in kinase activities often result in dramatic changes in these processes. Importantly, deregulated kinases are frequently reported as oncogenic, driving the overwhelming growth and spread of cancer cells. The human genome encodes for more than 500 protein kinases, which differ in substrate specificity and localization in cellular compartments [1]. Among the cytoplasmic kinases that possess specificity for serine/threonine residues, there are the protein kinases CK1α and CK2. Firstly, CK1α and CK2 were identified for the ability to phosphorylate casein in vitro [2]. Afterwards, casein kinase became a misnomer, since casein is not a physiological substrate of these two kinases [3]. To date, CK1α and CK2 share most of the features regarding the mechanism of action and the roles in the biology of healthy and diseased cells. Indeed, CK1α and CK2 are highly conserved within eukaryotes, ubiquitously expressed in vertebrates, pleiotropic, and constitutively active. Furthermore, CK1α and CK2 are described as players in cancer of different tissue origin [4,5,6]. In this review, we will focus on their role in cancer of blood origin, since it has been demonstrated that CK1α and CK2 are overexpressed and overactive in acute and chronic leukemias, non-Hodgkin lymphomas (NHL), and in multiple myeloma (MM) [7,8,9,10,11,12,13,14]. Different to other protein kinases, the “gain of expression/function” of these two kinases is poorly associated to genetic alterations, which are rare. Actually, these two kinases are outside the paradigm of oncogene addiction. Instead, the current view suggests that cancer cells’ dependency on these kinases resembles the phenomenon of “non-oncogene” addiction [15,16,17]. In this review, we will summarize the general features of CK1α and CK2, followed by their impact on some tumors of blood origin. In particular, we will illustrate (1) the most relevant oncogenic signaling axes regulated by CK1α and CK2 phosphorylation, which drive proliferation and survival; (2) how CK1α and CK2 contribute to sustain the stress experienced by blood cancer cells; and (3) the current evidence, which indicates that these two kinases could be part of the multi-therapeutic approach in hematological malignancies. 

## 2. General Features of CK1α and CK2: Structure, Regulation, and Function 

### 2.1. CK1α 

CK1α is one of the seven isoforms (α, β, γ1, γ2, γ3, δ, and ε) of the CK1 serine/threonine kinases family, whose members share homology in the N-terminal kinase domain and similar substrate preferences [18].

CK1α is encoded by the *CSNK1A1* gene located on chromosome 5q32 and four alternative spliced transcript variants are attested. CK1α acts as a “phosphate-directed” kinase, whose targeting is primed by a single phosphorylated side chain at position n−3 or n−4 relative to the serine/threonine (canonical motif) or non-canonical motif of pS/T-X- pS/T [19]. CK1α is a monomeric enzyme that has been generally found in a constitutive active state and ubiquitously expressed [20]. The ubiquity mirrors the involvement of this kinase in diverse signaling pathways, spanning from cell cycle progression to apoptosis, autophagy, circadian rhythm, DNA damage, and stress response. Among the many roles, CK1α is one of the main players of the Wnt/β-catenin signaling and targets p53 by different means [4]. 

Jiang et al. [5] extensively reviewed the CK1α expression data in normal and cancer human tissues, through RNA sequencing databases, revealing that the mRNA expression of the protein kinase has an important prognostic value in some cancer types [5]. In hematological cancers, CK1α has been found overactive in myeloid and lymphocytic leukemias [21], in diffuse large B-cell lymphoma (DLBCL) [22], and in MM [23,24]. However, the loss of CK1α in del(5q) myelodysplastic syndromes (MDS) has a different role with respect to the aforementioned blood malignancies [25]. 

### 2.2. CK2

In humans, protein kinase CK2 exists as a tetrameric enzyme, consisting of two catalytic subunits (CK2α and/or CK2α′, encoded by the *CSNK2A1* and *CSNK2A2* genes, respectively) and two regulatory CK2β subunits (encoded by the *CSNK2B* gene). The α subunits harbor the active site between the C- and N-terminal domains, while the C-terminus of the β subunits contacts the catalytic subunits to enhance and stabilize the enzymatic activity. The regulatory β subunit participates in the assembly of tetrameric complexes and in the modulation of CK2 substrate selection. However, the free catalytic CK2α subunits can independently phosphorylate substrates [26]. The consensus motif recognized by CK2 consists of the S/T proximal to acidic amino acid S/T-X-X-Ac, where Ac is an acidic amino acid [26]. CK2 is not subjected to absolute “on/off” regulation and thus it was found as a constitutive active enzyme, even in the absence of the regulatory CK2β subunit. Finally, CK2β was found to bind a number of proteins in the absence of catalytic CK2 subunits, such as A-Raf, c-Mos, and Chk1 serine/threonine protein kinases, modulating the activity of the enzymes [27].

CK2 is involved in a broad spectrum of biological processes, such as cell proliferation, differentiation, and apoptosis, by dynamic interplay with other constituents of the signaling network [28]. Indeed, CK2 is a player in more than 20% of the phosphoproteome [29]. CK2 is expressed in all organisms and tissues and it is essential for normal embryo development, as witnessed by the lethality of the CK2β knockout mouse [30]. CK2 expression is higher in a variety of solid tumors compared to normal tissues/cells, a repertoire which is in expansion [31]. This kinase acts by synergizing with oncogenes and boosting cancer hallmarks. This evidence is also reported in hematological malignancies. Indeed, the kinase has a pivotal role in mature and stem cell biology in acute leukemias [32,33]. It is overactive and overexpressed compared to healthy B-cells in chronic lymphocytic leukemia (CLL) [8]. Finally, in MM and NHL primary samples and cell lines, the α and β subunits were found overexpressed, regulating survival and proliferative signatures [34,35,36].

## 3. Protein Kinases CK1α and CK2 in Hematological Malignancies 

Distress or abnormal activation in kinase and phosphatase activities are either a driver or a direct consequence of diseases, especially in cancer [37]. These events influence signaling pathways and propel many of the aberrant hallmarks of cancer [38]. Kinases become uncontrolled following genetic and epigenetic alterations [37]. In blood tumor biology, tyrosine and serine/threonine kinases are frequently dysregulated by oncogenic mutations [39,40,41], and from a therapeutic standpoint, inhibition of protein phosphorylation has been successfully employed. The first example is the use of the inhibitor imatinib mesylate in chronic myeloid leukemia (CML), which targets the aberrant kinase activity of BCR-Abl1 [42]. Along this line, other kinases inhibitors, such as BTK (ibrutinib, acalabrutinib, and zanubrutinib) and PI3K (idelalisib, duvelisib, copanlisib, and others), have been developed among the therapeutic opportunities for blood malignancies [43,44,45]. Nevertheless, several mechanisms of resistance exploited by cancer cells have been reported [37]. 

Mutations occurring on CK1α and CK2 encoding genes, which could justify the overexpression/hyperactivation in cancers of blood origin, are very rare or not reported. The *CSNK1A1* somatic mutations occurring on the non-deleted allele of del(5q) MDS patients represent an exception [25,46]. Recently, Liu et al. have reported that the recurrent hot-spot mutations E98K and D140 in CK1α may confer a survival advantage and increased tumorigenic potential in in vitro studies [47]. Deletions of the *CSNK2B* gene were reported in DLBCL [48]. So far, only a very low percentage of cases of adult T acute lymphoblastic leukemia (ALL) and T lymphomas were found affected by indels and non-sense mutations in *CSNK1A1*, *CSNK2A1*, and *CSNK2B* genes [49]. Very recently, a first case of the *CSNK2A1* fusion gene in cancer was reported [50]. By RNA-sequencing methods, the authors identified the fusion gene *CSNK2A1-PDGFRB* in a patient with myeloid neoplasm with eosinophilia. When transduced in the cell line BaF3, the fusion protein was constitutively active, rendering the cells highly sensitive to imatinib treatment [50].

These features ascribe a peculiar contribution of CK1α and CK2 in tumorigenesis, which falls in a new paradigm [15]. 

### The Importance of Being “Non-Oncogene”

The aberrant phenotype of cancer cells (hallmarks of cancer) relies on the activity of specific driver alterations of oncogenes and related pathways [38]. The cost of the tumor progression/raising is the re-wiring of many processes, amongst them the augmented cellular stress levels, such as increase in DNA damage and replication stress, metabolic, proteotoxic, and oxidative stress [51]. To cope with stress, tumor cells might rely on the constitutive function of genes that are not oncogenic per se, not mutated, or aberrantly expressed. Importantly, these genes and pathways are essential to support the oncogenic phenotype of cancer cells but are not required to the same degree for the viability of normal cells [15,52]. This means that cancer cells became addicted to oncogenes [53] and non-oncogenes to sustain disease progression [15]. CK2 behavior fits with this phenomenon, which is broadly reported in the literature [16,52]. Indeed, the CK2 levels were found higher in malignant cells than in normal cells of the same type and its constitutive activity is often related to an anti-apoptotic and pro-survival effect. On one side it enhances the activity of the oncogenes, and on the other side it down counteracts tumor suppressors. Finally, CK2 protects the onco-kinome, controls multidrug resistance, neovascularization and inflammation [16,52]. In summary, CK2 functional activity covers almost all the hallmarks of cancer. 

Even if CK1α is not as directly associated with non-oncogenic cancer addiction as CK2, it owns the feature of a “conditionally essential malignant gene” [22,54]. 

To conclude, these two kinases rarely have been found mutated in tumors and their roles are strictly related to cellular fitness and stress resilience. Therefore, cancer cells are strongly sensitive, even to a small reduction in kinase function [55].

## 4. CK1α and CK2 Sustain Proliferation and Survival, and Counteract Apoptosis 

Beyond the genetic and epigenetic alterations implicated in cancer cell biology, tumor cell survival and clonal expansion rely on the dysregulation of a complicated signaling network, which fuels and sustains cancer progression. Many blood tumors are characterized by the hyperactivation of signaling cascades such as NF-κB, PI3K/AKT, STAT3, RAS-MAP kinase, Wnt/β-catenin, and others, which are responsible for the cancer phenotype. This “hyperactivity” creates an advantage for the cancer cell, supporting the tumor selection and clonal evolution. In the next paragraphs, we will depict the contribution of protein kinases CK1α and CK2 in the modulation of proliferative and survival pathways overactive in hematological malignancies, which are summarized in Table 1.

### 4.1. PI3K/AKT

The PI3K/AKT pathway is often found dysregulated in hematological malignancies [56]. AKT-mediated signaling is modulated by upstream PTMs exerted by other kinases such as PI3Ks kinases and mTOR, which phosphorylates AKT on Ser473. The tumor suppressor PTEN indirectly terminates AKT signaling by decreasing the cellular content of the second messenger PIP3. CK2 is also an AKT regulator, suggesting multiple levels of crosstalk [57]. Indeed, CK2 activates AKT by a direct phosphorylation on Ser129 [58]. Furthermore, CK2 regulates AKT in an indirect fashion, by phosphorylating PTEN on a cluster of C-terminal residues (Ser370, Thr366, and Ser385). This event stabilizes PTEN to proteasomal degradation, inhibiting PTEN phosphatase activity [59]. In adult B-ALL cells, the PI3K/AKT pathway is overactive despite an unexpectedly high expression of PTEN. In this context, high CK2 expression lowered the activity of PTEN with respect to healthy controls, phenotyping the loss of PTEN. Accordingly, B-ALL cells challenged with the CK2 inhibitor CX-4945 restored the PTEN activity and exerted a decrease in PI3K/ATK pathway activity [60]. In a similar fashion, in CLL, the apoptosis observed upon CK2 inhibition correlated with increased PTEN activity [8,61]. On leukemia stem cells (LSCs), CK2 also modulates AKT impinging on Ser473 phosphorylation, thus affecting stem cell viability and proliferation [33]. 

Protein kinase CK1α regulates the AKT pathway acting on the mTOR node. Indeed, CK1α mediates the phosphorylation of the mTOR inhibitor, DEPTOR, on Ser286, Ser287, and Ser291, to address the protein towards proteasomal degradation, thus activating the mTOR-mediated survival cascade. Accordingly, pharmacological inhibition of CK1 increases the DEPTOR levels and inhibits mTOR signaling. This effect could be relevant for cancers with high mTOR activity [62]. Similarly, in MM, CK1α inactivation caused a reduction in the phosphorylated AKT on Ser473 and of the total AKT protein level [24].

### 4.2. NF-κB

The NF-κB transcription factors family is a coordinator of inflammatory response, cell differentiation, proliferation, and survival, driving the transcription of several target genes. Both canonical and non-canonical NF-κB pathways have been implicated in hematological malignancies, especially in lymphoid leukemias and lymphomas, where the uncontrolled/constitutive activation of NF-κB results from a great variety of mechanisms [63]. Actually, mutation of NF-κB are rare and deregulated activation is frequently due to oncogenic signals, which trigger the constitutive activity of upstream pathways, leading to enhanced NF-κB transcriptional activity [64]. 

CK1α has a dual role in the regulation of NF-κB. It initially triggers T-cell receptor-induced NF-κB signaling by associating with the activating CBM1 (CARD11/BCL10/MALT1) complex. On the other side, by phosphorylating CARD11 on Ser608, it exerts a negative influence on the pathway, terminating the signaling [22]. Recently, it has been reported that CK1α phosphorylates also MALT1 on Ser562 in vitro [65]. Globally, CK1α triggers the formation of the CBM complex and subsequent activation of downstream cascades, supporting cell viability of NF-κB-addicted ABC-DLBCL lymphoma cells [22,65].

CK2 was shown to influence NF-κB activation by a dual mechanism. CK2 phosphorylates IkBα at Ser283, Ser289, and Tyr291 and, in turn, facilitates its ubiquitin–proteasome-dependent degradation, promoting the transcription of NF-κB target genes [66]. A further mechanism of regulation consists of the direct phosphorylation of the NF-κB family member RelA/p65 on Ser529, empowering its DNA transactivation activity [67]. In MM and mantle cell lymphoma (MCL), the overexpression of CK2 has been correlated with high levels of phosphorylated p65 on Ser529. This phosphorylation event seems to be counteracted when CK2 is inhibited both pharmacologically and by RNA interference tools, as demonstrated by the drop in expression of some NF-κB target genes [35]. Furthermore, in Burkitt’s lymphoma (BL) and DLBCL cells lines, chemical inhibition of CK2 resulted in the downmodulation of phosphorylated RelA, reducing cell growth [36]. In B-ALL, the inactivation of CK2 in combination with bortezomib exerted a pro-apoptotic activation of NF-κB [68]. The supportive role of CK2 on NF-κB activation was also reported on LSCs, where CK2 inactivation downmodulated RelA/p65 Ser529 phosphorylation and cooperated with the NF-κB inhibitor BAY 11-7082 to induce cell death [33].

### 4.3. Wnt/β-Catenin Pathway

The Wnt/β-catenin signaling pathway is involved in many developmental and cell proliferation processes in both embryonic and mature organisms. Mutations in the Wnt/β-catenin pathway components were found in diverse human cancers. In the canonical Wnt/β-catenin cascade, all CK1 family members are involved. CK1α is an essential component in the pathway. In absence of the Wnt ligand, the kinase interacts with Axin and phosphorylates β-catenin (on Ser45), thus priming it for further phosphorylation by GSK3β and subsequent proteasomal degradation [69,70]. Thus, CK1α acts as a negative player in Wnt/β-catenin signaling. In Wnt/β-catenin-dependent tumors, such as colorectal cancer, molecules that activate CK1α could be beneficial [71]. MM strongly rely on the Wnt/β-catenin pathway for cell growth and expansion. Importantly, the Wnt ligand is provided in a paracrine manner in the bone marrow microenvironment [72]. Surprisingly, in MM, CK1α loss of function, both by pharmacological inhibition and by RNA interference approaches, caused a downregulation of both β-catenin phosphorylation on Ser45 and of total β-catenin protein (with a mechanism that could involve p53 and caspase), leading to MM cell apoptosis and growth arrest [24]. 

CK2 function is required for Wnt secretion and actively supports the Wnt/β-catenin pathway in different fashions [73,74]. CK2 phosphorylates Dvl [75] and β-catenin [76,77], stabilizing β-catenin and recruiting Wnt regulators to the target genes [78]. In CLL, CK2 chemical inhibition with CX-4945 reduced the Dvl- and β-catenin-related gene expression [79]

### 4.4. JAK/STAT Signaling 

The JAK/STAT pathway is a key regulator in the signaling downstream cytokines. Activation of the STAT proteins, especially STAT5 and STAT3, are important in the pathogenesis of lymphoid and myeloid malignancies [80]. 

A cross-talk between the JAK/STAT proteins and CK2 was described, identifying CK2 as an activator of the signaling. Actually, CK2 constitutively associates with JAK1 and JAK2. The inhibition of this association caused a reduction in downstream STAT5 phosphorylation and subsequent activation of the apoptotic pathway. Evidence for this has been reported for chronic myeloproliferative disease (CMD), which suggested CK2 participation in tumors with constitutive JAK/STAT signaling [9,81]. After this first report, CK2 and JAK/STAT cross-talk was also reported in other hematological malignancies. CK2 inhibition in MM was accompanied with a reduction in STAT3 activation. In particular, CK2 inhibition rendered MM cells likely irresponsive to IL-6 elicitation by the reduction of STAT3 phosphorylation on Ser727. Furthermore, STAT3-dependent gene expression, namely *cyclin D1* and *IL-6* transcription, was compromised by CK2 pharmacological inhibition [35]. 

In CLL cells, STAT3 is constitutively phosphorylated on Ser727, affecting the transcription of genes important for cell survival [82]. Rozovski et al. [83] reported that a protein complex consisting of CK2, CD5, and BLNK induced phosphorylation of STAT3 on Ser727. Finally, in leukemia-initiating cells, CK2 exerts its role in cell survival, impinging on STAT3 phosphorylation [33]. 

## 5. Supportive Role of CK1α and CK2 in the Cancer Stress Phenotype

The higher proliferative rate and the increasing demand of protein synthesis in cancer cells could lead to the accumulation of unfolded/misfolded proteins. Indeed, proteotoxic stress is often experienced by tumor cells. To restore proteostasis, a prompt unfolded protein response (UPR) can be originated by the endoplasmic reticulum (ER) sensors (namely, PERK, ATF4 and IRE1α). UPR is a “double-edged sword”, since it may switch from a cytoprotective response to a pro-apoptotic one if the stress lasts/persists. Several reports have suggested that, mostly in solid tumors, cancer cells may take advantage of the UPR to cope with stress. Among the hematological malignancies, MM cells are high protein-producing/secreting cells; therefore, UPR is pivotal for survival [84]. In recent years, this adaptive response has emerged as a key player also in acute leukemias [85]. Several studies reported that CK2 controls ER/UPR signaling by protein phosphorylation or by regulating the transcription factors involved in ER stress/UPR signaling [86,87]. 

Together with ER response, autophagy is another evolutionary mechanism active also in cancer cells. As UPR, autophagy may be protective or deleterious for cancer cells, especially in hematological malignancies [88].

In the next paragraphs we will discuss CK1α and CK2 dependent modulation of signaling pathways involved in the cancer stress phenotype, which are summarized in Table 2.

### 5.1. Proteotoxic Stress

To overcome the ER engulfment, in some malignancies of blood/lymphoid origin, CK2 has been shown to participate in sustaining the proteotoxic/unfolded protein stress. In MM and other malignancies, tumor cells are frequently addicted to chaperones like Hsp90, which is a major player in the UPR [89]. CK2 is part of a “trinity” together with the chaperones Hsp90 and Cdc37. CK2 phosphorylates Cdc37 on Ser13 [90], strengthening the association with Hsp90 and subsequently with client protein kinases [91,92]. The CK2-dependent phosphorylation site is essential for the binding of Cdc37 to kinases, including AKT, RAF, and ERK, which are typically overexpressed in cancer. In a panel of NHL cell lines, the pharmacological inhibition of CK2 revealed a marked downmodulation of the phosphorylation on Ser13 on the chaperone Cdc37 [36]. 

In MM, under ER stress induced by thapsigargin, CK2 localized in the ER, increasing its catalytic activity. CK2 inactivation in MM cells influenced the homeostatic molecules regulating ER stress and UPR. Indeed, the co-chaperones Bip/Grp78 and the kinase/endoribonuclease IRE1α decreased, while an increase in phosphorylation of PERK and the initiation factor EIF2α was detected in Thr981 and Ser51, respectively. These lines of evidence suggest that CK2 might inhibit the branch of UPR that led to apoptosis [89]. A similar outcome was observed in T-ALL and B-ALL cells upon CK2 pharmacological inhibition. The perturbation of UPR emerged through the upregulation of Bip/Grp78 and the final switch towards apoptosis via upregulation of CHOP [68,93]. 

Overall, the alteration of UPR response, through CK2 inhibition, led to strong apoptosis. 

### 5.2. DNA Damage Stress 

CK1 family members and CK2 are involved in DNA damage repair and related signal transduction [4,94]. In the context of DNA damage-associated signaling, the tumor suppressor p53 is activated to ensure genomic stability. PTMs, such as p53 N-terminal phosphorylation, modulate the p53 response. The CK1 family members, such as α, δ, and ε, are able to phosphorylate p53 in multiple N-terminal sites (Ser6, Ser9, Ser15, Thr18, and Ser20) [95]. CK1α directly phosphorylates (on Ser20) and activates p53 upon HHV-SB viral infection [96]. The biological significance of the other direct phosphorylations exerted by CK1α are still unknown and only hypothesized.

The CK1α interaction with both the p53 inhibitors MDM2 and MDMX is well characterized. Inhibition or depletion of CK1α limits the association with MDM2, leading to p53 stabilization [97,98]. Phosphorylation on MDMX Ser289 by CK1α increases its association with p53, empowering its p53 inhibition capability [99]. Upon DNA damage, the interaction of CK1α–MDMX is disrupted with the promotion of p53 activation [100].

In MM, CK1α inhibits p53 and prevents the caspase-mediated degradation of AKT and β-catenin, promoting a cell-survival advantage [24]. In AML, knockdown or downregulation of CK1α correlates with increased expression of a p53 signature [21], and a more recent study reports that the blockage of p53 by CK1α prevents autophagy activation [101].

CK2 is involved in the repair both of single-strand [102] and double-strand DNA breaks [103,104,105]. Among other substrates, CK2 phosphorylates proteins such as MDC1 and XRCC1, which are part of complexes with other proteins responsible of the DNA damage response. Accordingly, in vitro studies combining pharmacologic inhibition of CK2 with DNA-targeted chemotherapeutic agents (gemcitabine and cisplatin) decreased the phosphorylation of MDC1 and XRCC1, leading to accumulation of double-strand and single-strand DNA breaks, as demonstrated in ovarian cancer cells [106]. Overall, CK2 is a key regulator in DNA damage response and facilitates the recruitment of proteins to promote DNA repair. Although in hematological malignancies the CK2 protection from DNA strand breaks is still unexplored, a role of CK2 in these pathways can be envisioned.

Upon UV-induced DNA damage, CK2 phosphorylates p53 on Ser392, resulting in enhancement of p53 activity [107]. Moreover, CK2 phosphorylates MDM2 at multiple sites (Ser260, Ser267, and Ser269) [108,109]. The phosphorylation at Ser267 slightly reduces the ability of MDM2 to direct p53 degradation [108]. CK2 phosphorylates the ubiquitylation enzyme USP7/HAUSP on Ser18, promoting MDM2 stabilization and therefore p53 inhibitions [110]. In CK2α transgenic mice, p53 deficiency collaborates with CK2 overexpression to promote lymphoma induction [111]. In AML cell lines, CK2α overexpression was accompanied by a p53 decrease. Conversely, CK2 inhibition was followed by an accumulation of p53, confirming the involvement of CK2 in the oncosuppressor p53 activity [112,113]. 

### 5.3. Autophagy

In general, CK1α is associated with a negative regulation of autophagy [5]. CK1α was described as a regulator of the autophagy pathway in RAS-driven cancers.

The loop consistent in RAS/PI3K/AKT/mTOR upregulated CK1α abundance, which in turn phosphorylates FOXO3A on Ser318 and Ser321. This phosphorylation event negatively influences the capability of FOXO3A to induce transactivation of pro-autophagic genes, blocking the autophagic flux. Depletion or pharmacological inhibition of CK1α enhanced autophagic flux in oncogenic RAS-driven cancers in human fibroblasts and multiple cancer cell lines [114]. In MM, the CK1α/δ dual chemical inhibitor D4476 reduced CK1α-dependent FOXO3A phosphorylation, increasing autophagic gene expression, but the accumulation of ineffective autophagic vesicles led to apoptosis. Upon CK1α gene silencing, even if an initial induction of autophagic flux was observed, the FOXO3a dependent transcriptional response was not achieved, resulting in autophagic flux breakdown and final apoptosis. These lines of evidence suggest that CK1α sustains a protective autophagy in the context of MM [115]. Recently, it has been reported that, in AML, CK1α exerts an opposite role in autophagy regulation, via the inhibition of p53/MDM2-mediated autophagy. Both pharmacological and genetic inhibition of CK1α induced autophagy and apoptosis in AML cell lines and patient blast cells [101]. Thus, the influence of CK1α on autophagy may be cell type and/or disease context dependent. 

Even if in hematological malignancies a role of CK2 in autophagy has not been reported yet, its involvement in autophagy and cancer energetics has been analyzed in other malignancies, as reviewed in [116]. Indeed, the CK2-dependent modulation of diverse autophagic signaling pathways includes regulation of metabolic/mitochondrial stress, via PTEN and mTOR. 

## 6. Therapeutic Strategies 

Therapeutic targeting of protein kinases has been demonstrated as the most promising approach in hematological malignancies [37]. Important is the inhibitor of aberrant kinase BCR-Abl1, imatinib mesylate in CML, and, more recently, other kinase inhibitors targeting proteins downstream of BCR in B-cell derived malignancies [43]. 

Since neoplastic transformation is often due to multiple causes/aberrations, the attention on a multitarget approach is raised. Given the multifaceted role of protein kinases CK1α and CK2 in MM, lymphomas, leukemia, and myeloid malignancies, the interest on targeting these kinases has been growing during the last years. 

Regarding CK1α, the development of selective inhibitors of CK1α is challenging, due to the high degree of homology among CK1 family isoforms [18]. So far, the pharmacological inactivation with the dual CK1α/CK1δ inhibitor D4476, exerted in cells from MM and other malignancies, produces a cytotoxic effect. This molecule has been extensively used to investigate CK1α biology. Very recently, the compound A-51, a dual inhibitor of CK1α and the kinase CDK7/9, has recently reached a Phase I clinical trial for the treatment of relapsed or refractory AML/high-risk MDS (clinical trial # NCT04243785). In the preclinical study, A-51 was the “lead” inhibitor among a series of novel compounds that were effective in CK1α inhibition and in induction of p53 activity and β-catenin upregulation in vivo [117]. Since the series of compounds inhibited also CDK7/9 activity, a synergistic pro-apoptotic activity due to the dual targeted pathways was observed [117]. 

An alternative approach to protein kinase CK1α inhibition could be the use of the immunomodulatory agent lenalidomide. Lenalidomide was reported to reduce CK1α expression in del(5q) MDS and MM [24,118]. The mechanism of action of lenalidomide in del(5q) MDS, where the *CSNK1A1* allele is lost, consists of the degradation of the residual CK1α via the E3 ubiquitin-ligase cereblon [119]. Notably, in MM, CK1α inactivation (with D4476 or RNA interference) synergizes with lenalidomide in inducing MM cell cycle arrest and apoptosis [24]. 

Regarding CK2, several compounds have been described, mostly focused on small molecules inhibiting kinase activity at the ATP-binding site. One of the first ATP-competitive CK2 inhibitors was DRB, which was followed by TBB and DMAT. Furthermore, derivatives of natural compounds, such as flavonoids (quercetin and apigenin), coumarin (ellagic acid), and anthraquinones (emodin and quinalizarin), were found with CK2 inhibitory activity. Finally, carboxyl acid derivatives comprise TBCA, CX-5011, and the clinical grade compound CX-4945 (silmitasertib) [120,121]. Due to the highly conserved nature of the ATP-pocket across kinases, the ATP competitive compounds suffer from off-target action. The highly selective CX-4945 inhibits other kinases, such as dual-specificity tyrosine phosphorylation-regulated kinase 1A (DYRK1A) and Cdc2-like kinases (Clks), in vitro, and affects alternative splicing in numerous genes [122,123,124]. 

Alternative strategies to inhibit CK2 have been explored by targeting the allosteric sites of CK2. Interestingly, the identification of the αD pocket near the ATP-binding site led to the development of more selective inhibitors [125,126]. Molecules DRB, W16, CAM187, and cyclic peptide Pc recognize the CK2α/β interface and antagonize the assembly of the CK2 holoenzyme complex [127]. Recently, it has been reported that aminothiazole derivatives, which recognize an alternative binding pocket in CK2, stabilize an inactive conformation of the enzyme [128]. The identification of allosteric/alternative sites offers the opportunity to design more selective compounds to target CK2 in anti-cancer treatments. 

However, the most effective and suitable compound has been proven to be CX-4945. Indeed, in vitro and in vivo studies have shown that CK2 inhibition with CX-4945 counteracts cell growth, by inducing apoptosis. Currently, CX-4945 has reached Phase I or II clinical trials in solid and blood tumors, such as cholangiocarcinoma, kidney cancer, basal cell carcinoma [129], and relapsed/refractory MM (clinical trial # NCT01199718).

In the literature, it is reported that CX-4945 enhanced the effect of different chemotherapeutic agents in MM, NHL, and acute and chronic leukemia. Indeed, CK2 blockage with CX-4945 boosted the effect of bortezomib (in B-T/ALL cell lines [68], MM and MCL [35]) and of the ER stress modulators (in MM [89]). Furthermore, CX-4945 modulated the AML sensitivity to the alkylating agent daunorubicin [113]. In CLL, the combination of fluradabine (a purine analogue inhibitor of DNA), ibrutinib, and idelalisib with CX-4945 improved the antiproliferative activity of the three molecules [130]. Furthermore, CX-4945 synergistically acted with the BCR signaling blockers, ibrutinib and fostamatinib, in inducing DLBCL cell death [131]. The multifunctional role of CK2 and effectiveness in in vitro studies provide the rationale for future use of CX-4945 in combination with other therapeutic drugs in the clinical scenario of leukemias (extensively reviewed in [129]). 

In summary, there is a body of data that supports the capability of CK1α and CK2 to act on the multiple hallmarks of cancer, suggesting that their inhibition could be envisioned as therapeutic strategy to target tumor cells at different levels. 

## 7. Conclusions

**Figure 1:**
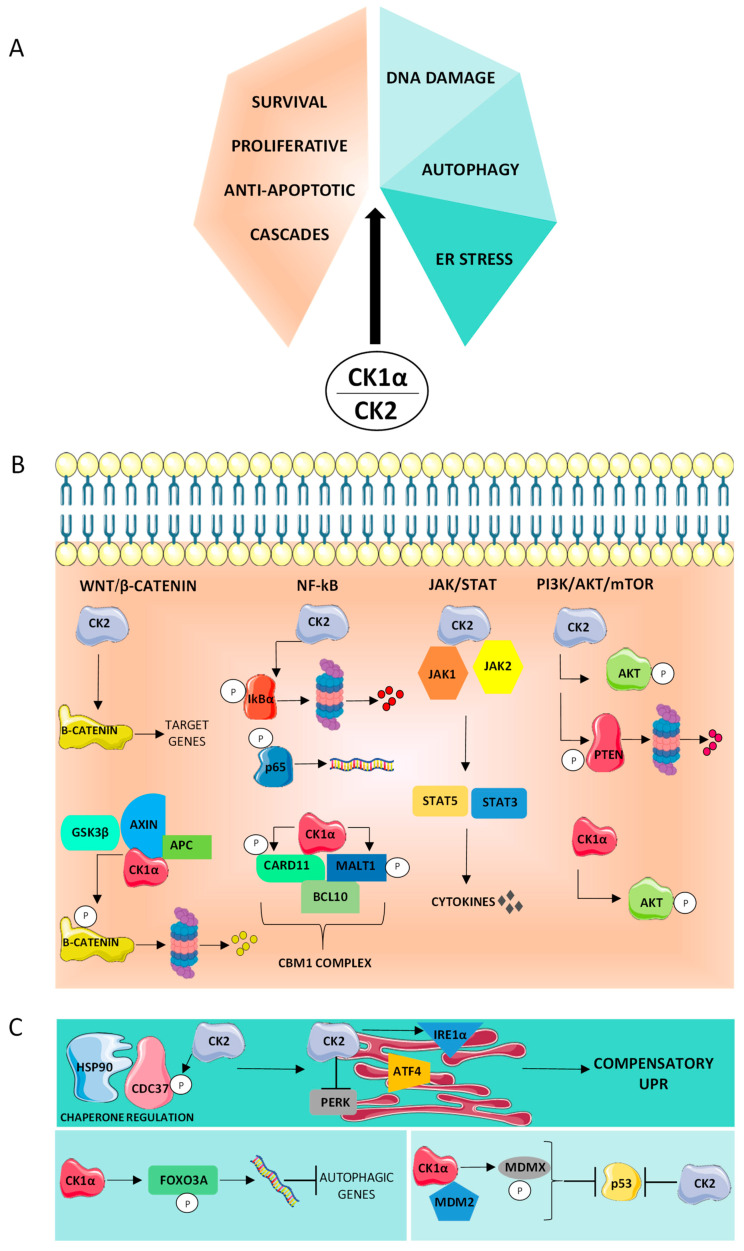
CK1α and CK2 regulate many cellular processes that sustain the tumor phenotype. (**A**) Summary of the biological processes controlled by CK1α and CK2 in blood cancers. (**B**,**C**) Dissection of the different cellular processes reported in (**A**). The same color code has been used in (**A**–**C**). (**B**) Role of CK2 and CK1α on the Wnt/β-catenin, NF-kB, JAK/STAT, and PI3K/AKT/m-TOR signaling pathways, which sustain cell survival and proliferation, counteracting apoptosis. Considering the Wnt/β-catenin pathway, CK2 sustains β-catenin dependent gene expression, while CK1α is responsible for the phosphorylation of β-catenin on Ser45, priming it for its subsequent proteasomal degradation. Focusing on NF-kB, CK2 directly phosphorylates both IkBα (on Ser283, Ser289, and Tyr291) and p65 (on Ser529), promoting the transcription of the NF-kB target genes. CK1α phosphorylates CARD11 (on Ser608) and MALT1 (on Ser562), regulating the CBM1 complex and NF-kB activation. In the JAK/STAT pathway, CK2 constitutively associates with JAK1 and JAK2, favoring STAT5 and STAT3 activation. Considering the PI3K/AKT/m-TOR cascade, CK2 directly phosphorylates AKT (on Ser129) and PTEN proteins (on Ser370, Thr366, and Ser385). Phosphorylated PTEN is degraded by a proteasomal mechanism, indirectly regulating AKT activation. CK1α sustains AKT expression and modulates the level of phosphorylated Ser 473 AKT. (**C**) Role of CK1α and CK2 in ER stress, autophagy, and DNA damage response. In ER stress (upper panel), CK2 phosphorylates Cdc37 on Ser13, increasing its association with Hsp90. CK2 influences the homeostatic molecules regulating UPR, in particular the PERK and IRE1α branches, promoting survival. Considering autophagy, (bottom left panel), CK1α phosphorylates FOXO3A (on Ser318 and Ser321), inhibiting autophagic gene transcription. In the DNA damage response (bottom right panel), the interaction of CK1α with the p53 regulators MDM2 and MDMX impairs p53 function. CK2 negatively regulates p53 expression and activation. The figure was drawn using Servier Medical art templates licensed under a Creative Common Attribution 3.0 Generic License. http://smart.servier.com/ accessed on 15/02/2021. The proteasome cartoon was created with BioRender.com.

Since the discovery of CK1α and CK2, their participation in the initiation and progression of cancer has become progressively clear. In healthy cells, these two kinases are ubiquitous and pleiotropic, thus participating in a large array of signaling and basic biological events. These multifaceted roles are reported in solid and hematological malignancies as well. Indeed, in tumors of blood origin, CK1α and CK2 modulate the phosphorylation and activation of several targets (Table 1 and Table 2), affecting crucial pathways linked to cancer-cell fitness and resilience. CK1α and mostly CK2 seem to sustain almost all the cancer hallmarks (Figure 1A), in a way that involves typically a non-oncogene addiction mechanism. Indeed, CK1α and CK2 are at the cross-road between pro-growth signals coming from the microenvironment and stress signals emerging inside the cell (Figure 1B,C). Through the phosphorylation of some transcription factors, CK1α and CK2 influence the activation of genes pivotal for survival and responsiveness to the high amount of stress experienced by cancer cells. Importantly, the control of Hsp90 machinery by CK2 and the consequent stabilization of the onco-kinome has a crucial importance for the enhancement of oncogene activity. CK1α multiple interaction with p53 has an essential significance in counteracting apoptosis. Other investigations are needed to clarify whether CK1α and CK2 activity could converge and integrate in the pathways that we described in this review. 

From a therapeutic standpoint, targeting these two kinases in hematological malignancies may offer the opportunity to improve the action of the currently used chemotherapeutic agents. Given the high potential of these two kinases in favoring cancer hallmarks, future studies will be needed to predict and look for other potential substrates recognized by these kinases to be targeted.

## Figures and Tables

**Table 1:** CK1α and CK2 modulate in a direct and indirect fashion the signaling that sustains proliferation and survival, and counteracts apoptosis, in blood tumors.

SIGNALLING	CK1α TARGETS	BLOOD TUMOR	CK2 TARGETS	BLOOD TUMOR
PI3K/AKT	AKT	MM	AKT/PTEN	B-ALL, CLL, LSCs, DLBCL
NF-κB	CARD11/MALT	DLBCL	RelA/p65	MM, MCL, DLBLC, BL; ALL
Wnt/β-catenin	β-catenin	MM	β-catenin, Dvl	CLL
JAK/STAT			JAK1/2, STAT3/5	CMD, MM, CLL, AML, ALL

**Table 2:** CK1α and CK2 modulate in a direct and indirect manner the signaling involved in stress phenotypes.

STRESS PHENOTYPE	CK1α TARGETS	BLOOD TUMOR	CK2 TARGETS	BLOOD TUMOR
ER STRESS/UPR			Cdc37/Bip/Grp78, IRE1α, PERK	MM, NHL, T/B-ALL
DNA DAMAGE	p53	MM, AML	p53	AML
AUTOPHAGY	FOXO3A	MM		

## Data Availability

Not applicable.

## Abbreviations

ALL	Acute Lymphoblastic Leukemia
AML	Acute myeloid leukemia
ATF4	transcription factor 4
ATF6	Activating Transcription Factor-6
BCR	B-cell receptor
CHOP	C/EBP-homologous protein
CK1	Casein kinase 1
CK2	Casein kinase 2
CLL	Chronic Lymphocytic Leukemia
CMD	Chronic Myeloproliferative disorders
CML	Chronic Myeloid Leukemia
DEPTOR	DEP domain-containing mTOR-interacting protein
DLBCL	Diffuse large B-cell Lymphoma
Dvl	Dishevelled
eIF2α	eukaryotic translation initiation factor 2α
ER	Endoplasmic reticulum
FL	Follicular Lymphoma
FOXO3A	Forkhead Box O3
Grp78/Bip	glucose regulated protein 78/Binding immunoglobulin protein
GSK3-β	Glycogen synthase kinase 3-β
Hsp90	Heat Shock Protein 90
IL-6	Interleukin-6
IRE1α	Inositol Requiring Enzyme 1
JAK	Janus associated kinase-signal
LSCs	Leukemic-stem cells
MCL	Mantle Cell Lymphoma
MDM2	Mouse Double Minute 2
MDMX	Murine Double minute X
MDS	Myelodysplastic syndrome
MM	Multiple Myeloma
mTOR	Mammalian target of rapamycine
NF-kB	Nuclear Factor kappa-light-chain-enhancer of activated B cells
NHL	Non-Hodgkin Lymphoma
PDGFR	Platelet-derived growth factor receptors
PERK	PKR-like ER kinase
PI3K	Phosphoinositide 3-kinase
PIP3	Phosphatidylinositol (3,4,5)-trisphosphate
PTEN	Phosphate and tensin homolog
PTM	Post translational modification
STAT3	Signal transducer and activator of transcription 3
UPR	Unfolded Protein response
